# Susceptible cell lines for the production of porcine reproductive and respiratory syndrome virus by stable transfection of sialoadhesin and CD163

**DOI:** 10.1186/1472-6750-10-48

**Published:** 2010-06-29

**Authors:** Iris Delrue, Hanne Van Gorp, Jan Van Doorsselaere, Peter L Delputte, Hans J Nauwynck

**Affiliations:** 1 Department of Virology, Parasitology and Immunology, Faculty of Veterinary Medicine, Ghent University, Salisburylaan 133, 9820 Merelbeke, Belgium; 2 Department of Health Care and Biotechnology, KATHO Catholic University College of South-West Flanders, Wilgenstraat 32, 8800 Roeselare, Belgium

## Abstract

**Background:**

Porcine reproductive and respiratory syndrome virus (PRRSV) causes major economic losses in the pig industry worldwide. *In vivo*, the virus infects a subpopulation of tissue macrophages. *In vitro*, PRRSV only replicates in primary pig macrophages and African green monkey kidney derived cells, such as Marc-145. The latter is currently used for vaccine production. However, since virus entry in Marc-145 cells is different compared to entry in primary macrophages, specific epitopes associated with virus entry could potentially alter upon growth on Marc-145 cells. To avoid this, we constructed CHO and PK15 cell lines recombinantly expressing the PRRSV receptors involved in virus entry into macrophages, sialoadhesin (Sn) and CD163 (CHO^Sn-CD163 ^and PK15^Sn-CD163^) and evaluated their potential for production of PRRSV.

**Results:**

Detailed analysis of PRRSV infection revealed that LV and VR-2332 virus particles could attach to and internalize into the CHO^Sn-CD163 ^and PK15^Sn-CD163 ^cells. Initially, this occurred less efficiently for macrophage grown virus than for Marc-145 grown virus. Upon internalization, disassembly of the virus particles was observed. The two cell lines could be infected with PRRSV strains LV and VR-2332. However, it was observed that Marc-145 grown virus infected the cells more efficiently than macrophage grown virus. If the cells were treated with neuraminidase to remove cis-acting sialic acids that hinder the interaction of the virus with Sn, the amount of infected cells with macrophage grown virus increased. Comparison of both cell lines showed that the PK15^Sn-CD163 ^cell line gave in general better results than the CHO^Sn-CD163 ^cell line. Only 2 out of 5 PRRSV strains replicated well in CHO^Sn-CD163 ^cells. Furthermore, the virus titer of all 5 PRRSV strains produced after passaging in PK15^Sn-CD163 ^cells was similar to the virus titer of those strains produced in Marc-145 cells. Analysis of the sequence of the structural proteins of original virus and virus grown for 5 passages on PK15^Sn-CD163 ^cells showed either no amino acid (aa) changes (VR-2332 and 07V063), one aa (LV), two aa (08V194) or three aa (08V204) changes. None of these changes are situated in known neutralizing epitopes.

**Conclusions:**

A PRRSV susceptible cell line was constructed that can grow virus to similar levels compared to currently available cell lines. Mutations induced by growth on this cell lines were either absent or minimal and located outside known neutralizing epitopes. Together, the results show that this cell line can be used to produce vaccine virus and for PRRSV virus isolation.

## Background

Porcine reproductive and respiratory syndrome virus (PRRSV) is a member of the family *Arteriviridae*, order *Nidovirales *[1,2] causing major economic losses in the pig industry worldwide [3]. PRRSV infection may result in reproductive failure in sows and is involved in the porcine respiratory disease complex (PRDC) [4-9].

*In vivo*, the virus infects a subpopulation of tissue macrophages [10-13]. *In vitro*, efficient PRRSV replication is only observed in primary pig macrophages (e.g. alveolar macrophages) [14], differentiated monocytes [15] or African green monkey kidney derived cells, such as Marc-145 [14,16]. Infection of macrophages, the natural host cell of PRRSV, occurs via a few similar but also different receptors compared to infection of Marc-145 cells [17]. PRRSV first attaches to macrophages via heparan sulphate [18], then the virus is internalized via sialoadhesin (Sn) [19]. CD163 is also involved in infection of macrophages, probably at the stage of virus disassembly [20]. PRRSV infection of Marc-145 cells occurs via binding to a heparin-like molecule as a first step [21]. The nucleocapsid of PRRSV is described to bind to the intermediate filament vimentin, which is suggested to mediate transport of the virus to the cytosol [22]. CD151 may be involved in fusion of the viral envelope and the endosome, but the precise mechanism is yet unknown [23]. CD163 is also essential for PRRSV infection of Marc-145 cells, but its role in this process is still unclear [24].

Currently, PRRS vaccine virus is produced in Marc-145 cells. However, since virus entry in Marc-145 cells is different compared to entry in primary macrophages [25] and because adaptation is needed for growth on Marc-145 cells [26], it is possible that specific epitopes associated with virus neutralization are lost or modified. Although virus production in primary macrophages would be ideal to avoid adaptation, these cells cannot be used because of batch variation, risk of contamination with other pathogens present in the macrophages isolated from pigs and high production costs. Previous results in our lab showed that non-permissive cells transiently transfected with Sn only sustained internalization, but not infection [19]. Non-permissive cells transiently transfected with CD163 allow a low level of infection depending on the cell type used [24]. Co-expression of both Sn and CD163 is the most efficient for PRRSV infection in different cell lines evaluated [20].

To avoid the problems associated with PRRS vaccine virus production in other cell types, the aim of this study was to construct cell lines that recombinantly express Sn, the receptor that mediates PRRSV attachment to and internalization into macrophages [19,27] and CD163, which is most probably involved in virus disassembly in macrophages [20]. Both Sn and CD163 are needed to make a PRRSV susceptible cell line for virus production that mimics the natural entry pathway in macrophages.

## Methods

### Cells, viruses and plasmids

CHO-K1 cells were cultivated in F12 medium and PK15 cells in Dulbecco Modified Eagle Medium (D-MEM). Both media were supplemented with 10% fetal bovine serum (FBS), 2 mM L-glutamine, 1 mM non-essential amino acids, 1 mM sodium pyruvate and a mixture of antibiotics. The cells were maintained in a humidified 5% CO_2 _atmosphere at 37°C. Macrophages cultivated in medium containing RPMI-1640, 10% FBS, 2 mM L-glutamine, 1 mM non-essential amino acids, 1 mM sodium pyruvate and a mixture of antibiotics were used for titration. A pcDNA3.1D/V5-HisTOPO plasmid containing Sn cDNA and geneticine resistance gene [19] and a pBUD plasmid with CD163 cDNA and zeocin resistance gene were used for transfection. To construct the pBUD plasmid containing CD163, CD163 from a pcDNA3.1D/V5-HisTOPO plasmid containing CD163 [20], was cloned into a pBUD plasmid via restriction site HindIII and XbaI. The European prototype PRRSV strain Lelystad virus (LV), grown on Marc-145 cells and macrophages [14], the American prototype PRRSV strain VR-2332 grown on Marc-145 cells [26], and three recent Belgian isolates, belonging to the European type, grown on macrophages (07V063, 08V204 and 08V194) were used for inoculation.

### Transfection and selection

CHO-K1 and PK15 cells were transfected with FuGENE 6 (Roche) or Lipofectamine Plus (Invitrogen, Merelbeke, Belgium) respectively, according to the manufacturer's instructions. CHO-K1 and PK15 cells were first transfected with a plasmid containing the Sn cDNA and geneticine resistance gene. The cells were single cell cloned and selected for Sn expressing CHO and PK15 cells with geneticine (200 μg/ml, GIBCO). Afterwards, the obtained CHO^Sn ^and PK15^Sn ^cells were transfected with a plasmid containing the CD163 cDNA and zeocin resistance gene and single cell cloned. For the selection of CHO^Sn ^and PK15^Sn ^cells expressing CD163, zeocin (200 μg/ml, Invitrogen, Merelbeke, Belgium) was used.

### Screening of cells expressing Sn and CD163 by immunofluorescence staining

Transfected CHO-K1 and PK15 cells were fixed with methanol and stained with primary monoclonal antibodies (mAb) against Sn (mAb 41D3) [19,28] and CD163 (mAb 2A10, AbD Serotec) [29,30]. As a secondary antibody, fluorescein isothiocyanate (FITC)-conjugated goat polyclonal anti-mouse immunoglobulins (Invitrogen, Molecular Probes, Merelbeke, Belgium) were used. Screening of cells expressing Sn and/or CD163 was performed with a fluorescence microscope (Leica Microsystems GmbH, Heidelberg, Germany).

### Analysis of PRRSV infection of CHO^Sn-CD163 ^and PK15^Sn-CD163 ^cells by immunoperoxidase staining

CHO^Sn-CD163 ^and PK15^Sn-CD163 ^cells were seeded in 96-well plates and inoculated with virus. At different time points post inoculation (pi), the cells were fixed with methanol and an immunoperoxidase staining was performed [18]. Briefly, viral antigen positive cells were stained with primary mAb anti-nucleocapsid P3/27 [31] and secondary antibody peroxidase labeled goat anti-mouse immunoglobulin (Ig) (DakoA/S, Glostrup, Denmark). Afterwards, 3-amino-9-ethylcarbazole (AEC) substrate (Sigma, Bornem, Belgium) was added. The amount of infected cells was counted with a light microscope (Olympus Optical Co., Hamburg, Germany).

### Attachment, internalization, disassembly and infection of CHO^Sn-CD163 ^and PK15^Sn-CD163 ^cells with PRRSV analyzed by immunofluorescence staining

To determine attachment, internalization, disassembly and infection of CHO^Sn-CD163 ^and PK15^Sn-CD163 ^cells, the cells were seeded at 200 000 cells/mL and after 2 days of cultivation they were inoculated with virus. The cells were fixed with methanol after 1 hour incubation at 4°C to investigate attachment, since virus is not able to internalize at 4°C. The cells were fixed after 1 hour incubation at 37°C to determine the internalized particles. After 5 hours incubation at 37°C, the cells were fixed to analyze disassembly (disappearance of staining). To analyze infection, the cells were fixed after 24 hours incubation at 37°C. The virus was stained with a primary mAb anti-nucleocapsid P3/27 [31] and a secondary FITC-conjugated goat polyclonal anti-mouse immunoglobulins (Invitrogen, Molecular Probes, Merelbeke, Belgium). Virus particles were counted on images acquired with a TCS SP2 laser scanning spectrum confocal system (Leica Microsystems GmbH, Heidelberg, Germany).

### Virus production after passaging in CHO^Sn-CD163 ^and PK15^Sn-CD163 ^cells

CHO^Sn-CD163 ^and PK15^Sn-CD163 ^cells were seeded at 200 000 cells/mL in Tissue Culture (TC) flasks. After 2 days of cultivation, the cells were initially inoculated with 10^5 ^TCID_50 _of each virus strain. After 3 and 5 days post inoculation (dpi), the supernatant was collected and centrifuged for 30 min at 30 × g at 4°C. The supernatant was stored at -70°C and titrated. Virus titration was performed on 24 hours cultivated alveolar macrophages following the standard procedure [12]. After 3 days of incubation at 37°C, the occurrence of cytopathic effect (CPE) was investigated. Macrophages were fixed at 3 dpi and an immunoperoxidase staining was performed to identify infected cells. The 50% tissue culture infective dose (TCID_50_) was calculated.

### Virus sequencing after passaging virus in PK15^Sn-CD163 ^cells

RNA was extracted from PRRSV passaged 4 times on PK15^Sn-CD163 ^cells using a RNeasy Protect Mini Kit (QIAGEN) and reverse transcribed using random hexamers and MultiScribe Reverse Transcriptase (Applied Biosystems) according to the manufacturer's guidelines.

The primers ORF2a-FW (5'-gtsacaccktatgattacg-3') and ORF2a-REV (5'-tcatrccctattytgcacca-3'), ORF3-FW (5'-agcctacagtacaacaccac-3') and ORF3-REV (5'-agaaaaggcacgcagaaagca-3'), ORF4-FW (5'-cggcccaittccatccigag-3') and ORF4-REV (5'-cattcagctcgcataicgtcaag-3'), ORF5-FW2 (5'-tgcticatttcitgacacc-3') and ORF5-REV1 (5'-accttaagigcitatatc-3'), ORF6FW (5'-taccaactttcttctggac-3') and ORF6REV (5'-acccagcaactggcacag-3'), ORF7-FW (5'- tggcccctgcccaicacg-3') and ORF7-REV (5'- tcgccctaattgaataggtga-3') were used to amplify the different ORFs with Taq Polymerase (Invitrogen, Merelbeke, Belgium). PCR products were treated with Exonuclease I and Antarctic Phosphatase (New England Biolads, Ipswich, USA) and used directly for cycle sequencing with a Big Dye Terminator Cycle sequencing kit V1.1 (Applied Biosystems, Foster City, USA) and PRRSV primers. Cycle sequencing reaction products were purified by ethanol precipitation and separated on an ABI Genetic 310 (Applied Biosystems, Foster City, USA).

The sequences were analyzed and compiled by BlastN and BlastP http://www.ncbi.nlm.nih.gov, and Sixframe, ClustalW, Align (workbench.sdsc.edu). The Genbank accession number are for 07V063 [Genbank:GU737264], 08V204 [Genbank:GU737266] and for 08V194 [Genbank:GU737265].

## Results

### Construction of CHO^Sn-CD163 ^and PK15^Sn-CD163 ^cell line

**Figure 1:**
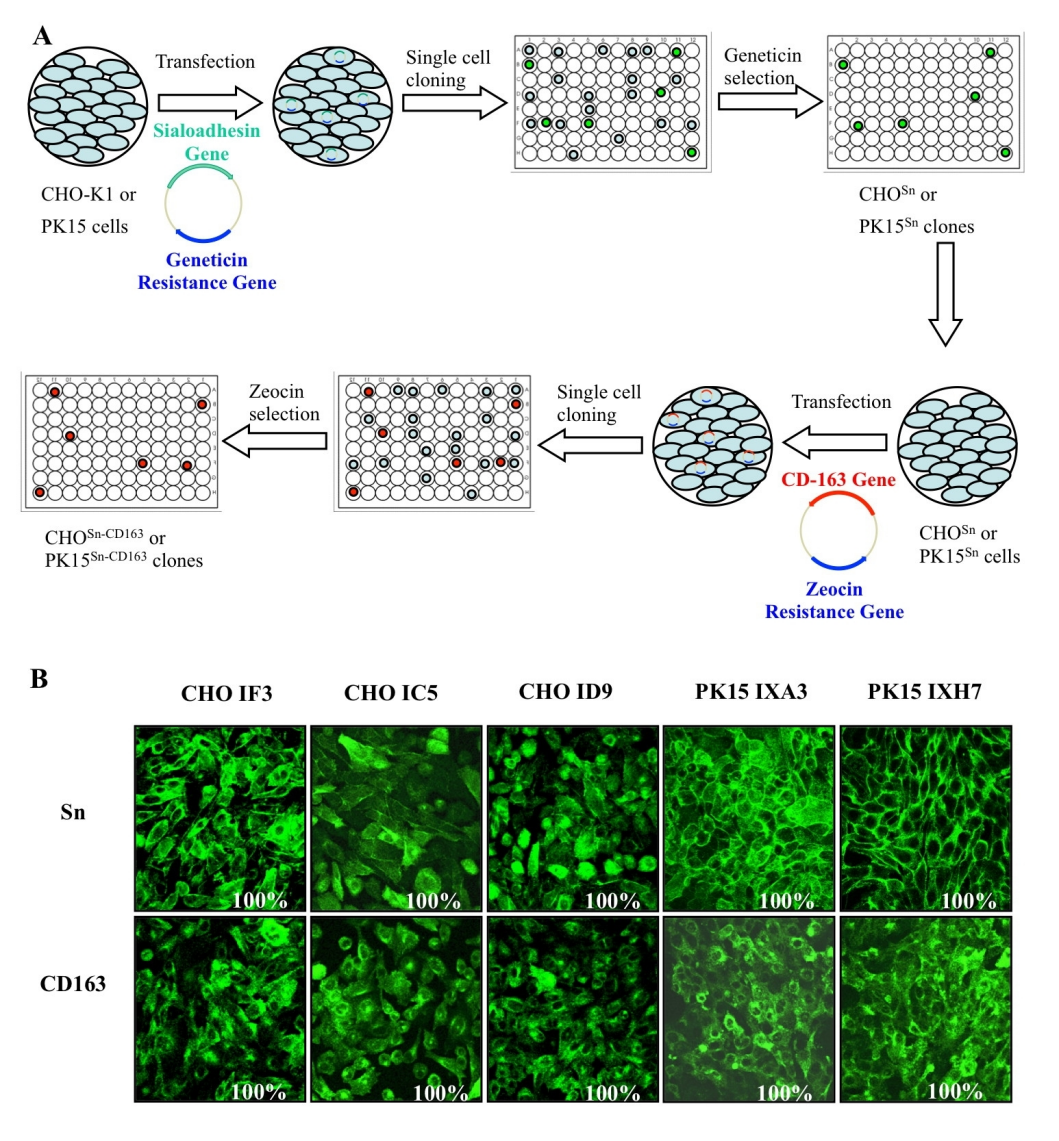
**Construction of CHO^Sn-CD163 ^and PK15^Sn-CD163 ^cell lines**. **A) Schematic representation of the construction of CHO^Sn-CD163 ^and PK15^Sn-CD163 ^cell lines** To construct a cell line co-expressing Sn and CD163, CHO-K1 or PK15 cells were transfected with a plasmid containing the Sn cDNA and a geneticine resistance gene. The cells were single cell cloned and clones were screened for Sn expressing cells. After selection for geneticine resistance, the obtained CHO^Sn ^or PK15^Sn ^cells were transfected with a plasmid containing the CD163 cDNA and a zeocin resistance gene, which allowed selection of cells expressing both Sn and CD163. B) Immunofluorescence staining of the obtained CHO^Sn-CD163 ^or PK15^Sn-CD163 ^cells for Sn and CD163. Some CHO^Sn-CD163 ^clones (IF3, IC5 and ID9) and PK15^Sn-CD163 ^clones (IXA3 and IXH7) are represented with their Sn and CD163 expression.

CHO-K1 and PK15 cells were transfected with Sn and CD163 and selected for cells expressing both receptors as shown in Figure 1A. The presence of Sn and CD163 was confirmed by immunofluorescence staining (Figure 1B). 16 CHO and 4 PK15 clones co-expressing Sn and CD163 (CHO^Sn-CD163 ^and PK15^Sn-CD163^) were selected. 10 CHO and 4 PK15 clones were obtained, in which 100% of the cells retained stable expression of Sn and CD163 for at least 15 passages. The other 6 CHO clones lost either Sn or CD163 expression after a few passages. After a preliminary screening for PRRSV susceptibility, 3 CHO clones (IC5, ID9 and IF3) and 2 PK15 clones (IXH7 and IXA3) were retained for further analysis.

### Effect of cell density and cultivation time of CHO^Sn-CD163 ^and PK15^Sn-CD163 ^cells on the susceptibility to PRRSV infection

To determine the effect of cell density and cultivation time of the cells on susceptibility to PRRSV infection, 3 CHO^Sn-CD163 ^cell clones (IC5, ID9 and IF3) and 2 PK15^Sn-CD163 ^cell clones (IXH7 and IXA3) were seeded at different cell densities (100 000, 200 000 or 300 000 cells/mL) and inoculated with 50 μL containing 10^4 ^TCID_50 _Marc-145 grown LV, Marc-145 grown VR-2332 or macrophage grown LV at different days post seeding (1, 2 or 3 days post seeding). After 2 dpi, the cells were fixed and stained.

**Figure 2:**
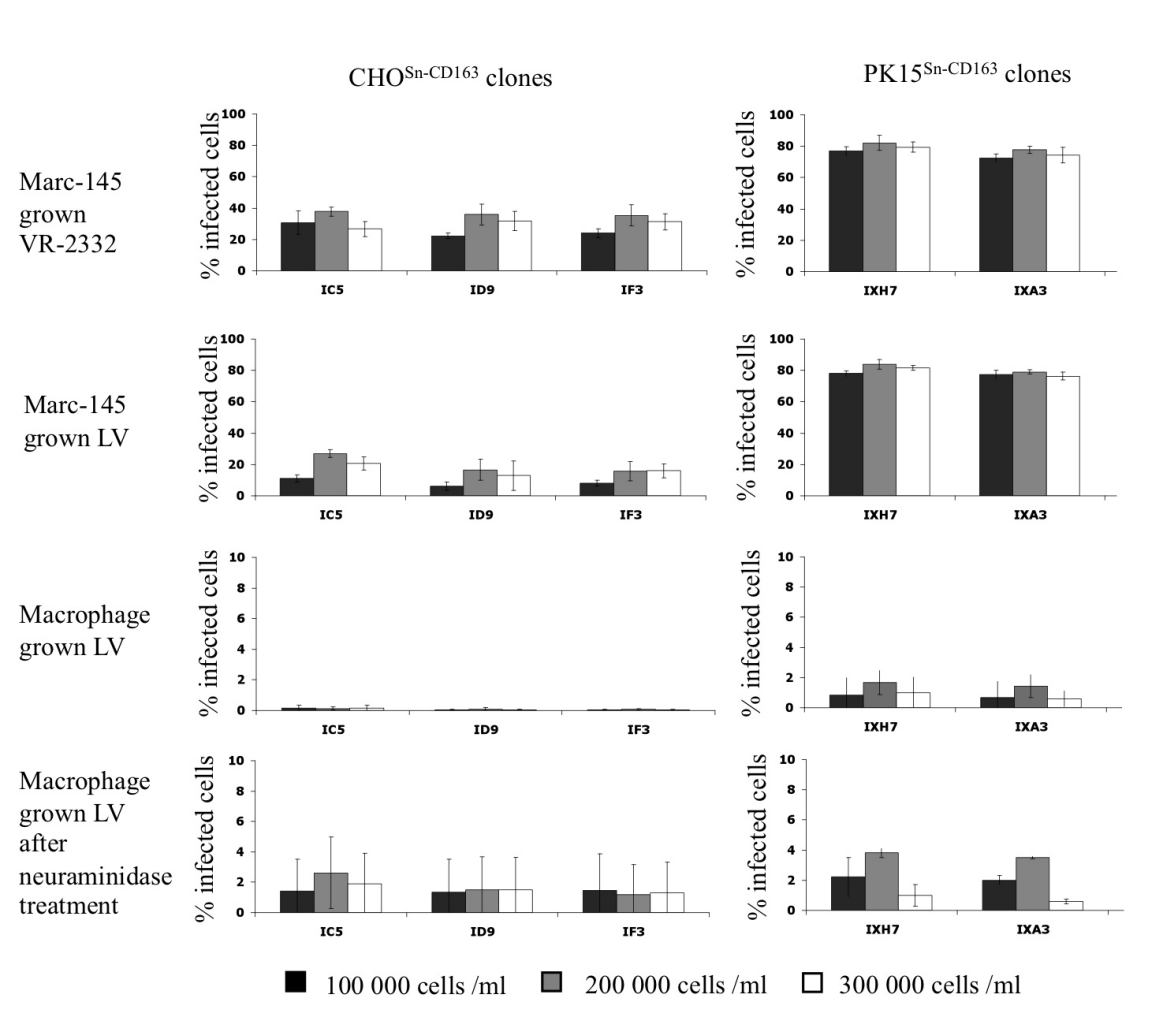
**Effect of cell density on susceptibility of CHO^Sn-CD163 ^and PK15^Sn-CD163 ^cells to PRRSV infection**. CHO^Sn-CD163 ^and PK15^Sn-CD163 ^cells were cultivated for 2 days before they were inoculated with Marc-145 grown LV, Marc-145 grown VR-2332 or macrophage grown LV. The black bars represent a cell density of 100 000 cells/ml, the grey bars 200 000 cells/mL and the white bars 300 000 cells/ml. The graphs show the percentage of infected cells. Values represent mean ± SD of three experiments.

For Marc-145 grown virus infection of CHO^Sn-CD163 ^cells, little difference was observed between different cell densities and days post seeding, although a density of 200 000 cells/mL and inoculation at 2 days post seeding seemed a little more efficient. The VR-2332 strain infected the CHO^Sn-CD163 ^cells more efficiently than the LV strain. For PK15^Sn-CD163 ^cells, the infection rate for Marc-145 grown LV as well as VR-2332 was approximately 80%, independently of densities and cultivation time. CHO^Sn-CD163 ^clones IC5, ID9 and IF3 were equally sensitive to virus infection. There was no difference in sensitivity for infection between PK15^Sn-CD163 ^clones IXH7 and IXA3. The PK15^Sn-CD163 ^cells could be infected more efficiently than the CHO^Sn-CD163 ^cells (Figure 2).

Both CHO^Sn-CD163 ^and PK15^Sn-CD163 ^cells showed a low infection rate with macrophage grown virus. The cell density had no influence on infection. There were no differences between clone IC5, ID9 and IF3 for CHO^Sn-CD163 ^and between IXH7 and IXA3 for PK15^Sn-CD163 ^cells (Figure 2). Overall, Marc-145 grown virus strains could infect the cells more efficiently than macrophage grown virus strains tested.

### Effect of neuraminidase treatment of CHO^Sn-CD163 ^and PK15^Sn-CD163 ^cells on the susceptibility to macrophage grown PRRSV infection

The previous section showed that macrophage grown PRRSV infection rate is very low. Previously, it was however shown that infection of macrophages can be enhanced after removal of sialic acid from the cells with neuraminidase, as observed in our lab [32]. Desialylation of macrophages enhances sialoadhesin-mediated lectin activity [33]. Since the interaction between sialic acids on PRRSV and sialoadhesin is important for infection of cells [34], desialylation of the CHO^Sn-CD163 ^and PK15^Sn-CD163 ^cells can probably enhance the amount of infected cells by macrophage grown virus. Therefore, to increase the infection rate of macrophage grown virus infection, 3 CHO^Sn-CD163 ^cell clones (IC5, ID9 and IF3) and 2 PK15^Sn-CD163 ^cell clones (IXH7 and IXA3) were seeded at different densities (100 000, 200 000 or 300 000 cells/mL) and were infected at different days post seeding (1, 2 or 3 days post seeding) with 50 μL containing 10^4 ^TCID_50 _macrophage grown LV, after treatment of the cells with neuraminidase to remove cis-acting sialic acids. After 2 dpi, the cells were fixed and stained. The results showed that treatment of the PK15^Sn-CD163 ^cells at 200 000 cell/mL with neuraminidase before inoculation enhanced infection of the cells with macrophage grown virus (Figure 2).

### Analysis of PRRSV attachment, internalization, disassembly and infection of CHO^Sn-CD163 ^and PK15^Sn-CD163 ^cells

**Figure 3:**
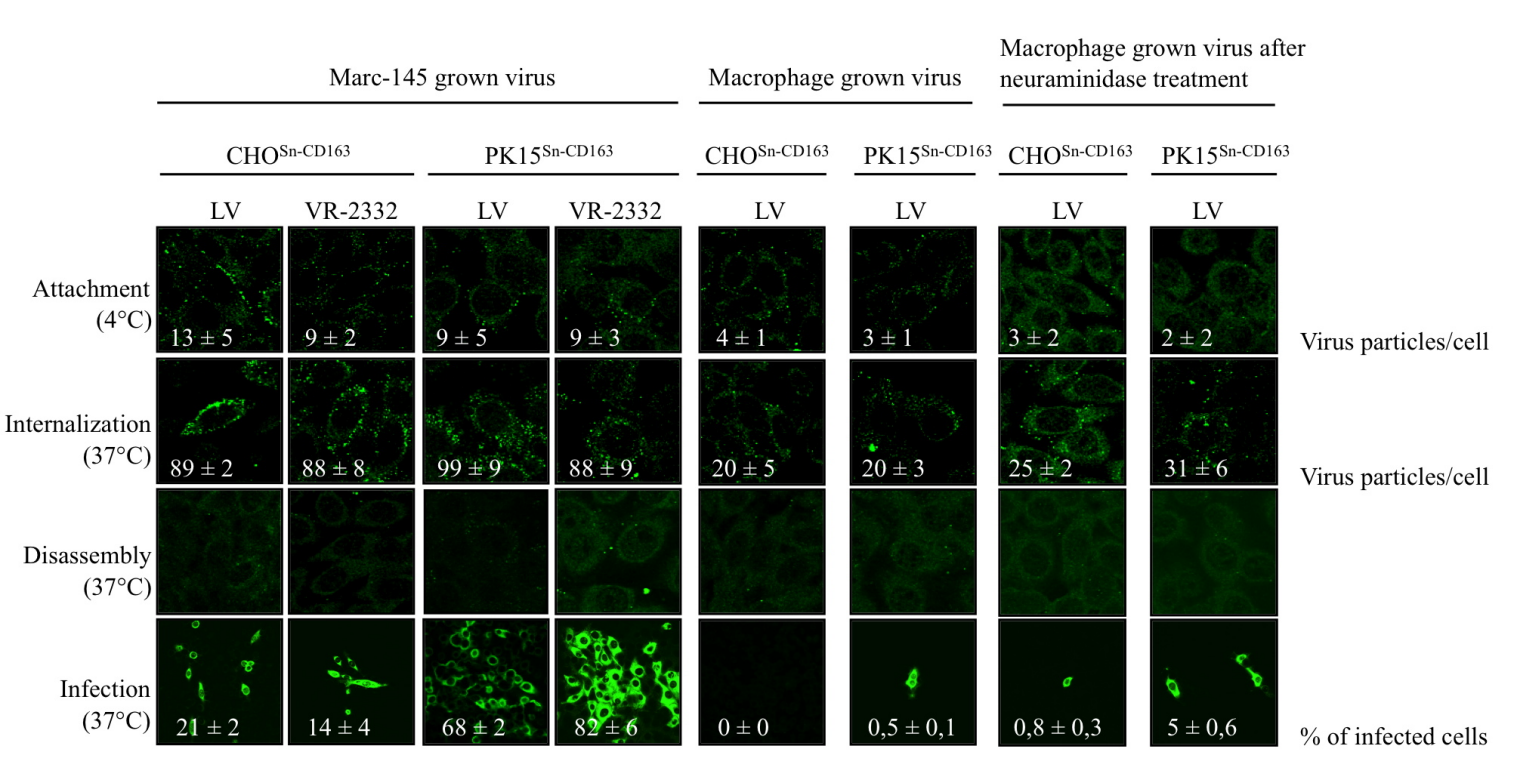
**Attachment, internalization, disassembly and infection in CHO^Sn-CD163 ^and PK15^Sn-CD163 ^cells**. CHO^Sn-CD163 ^clone IC5 and PK15^Sn-CD163 ^clone IXH7 were inoculated with Marc-145 grown LV, Marc-145 grown VR-2332 or macrophage grown LV. The different stages of the viral replication cycle were investigated by immunofluorescence staining of the virus.

To investigate if virus attachment, internalization, disassembly and infection occurs in the CHO^Sn-CD163 ^and PK15^Sn-CD163 ^cells, CHO^Sn-CD163 ^clone IC5 and PK15^Sn-CD163 ^clone IXH7 were inoculated with Marc-145 grown LV (moi 1), Marc-145 grown VR-2332 (moi 1) and macrophage grown LV (moi 1). The virus was stained by immunofluorescence at different stages of the viral replication cycle. First, the virus particles were clearly shown to attach to the cells. Then, virus particles were internalized into the cells, with macrophage grown virus being less efficient than Marc-145 grown virus. After internalization, the particles were uncoated to release the genome. Finally, infection occurred (Figure 3). In general, Marc-145 grown virus infected the cells more efficiently than macrophage grown virus. Further, Marc-145 grown VR-2332 infected the PK15^Sn-CD163 ^cells more efficiently than Marc-145 grown LV, while this was equal for the CHO^Sn-CD163 ^cells. Infection of the cells with macrophage grown virus was very low. Treatment of the cells with neuraminidase before inoculation enhanced the infection rate of PK15^Sn-CD163 ^cells. The PK15^Sn-CD163 ^cells were infected most efficiently in all cases (Figure 3).

### PRRSV infection kinetics on CHO^Sn-CD163 ^and PK15^Sn-CD163 ^cells

**Figure 4:**
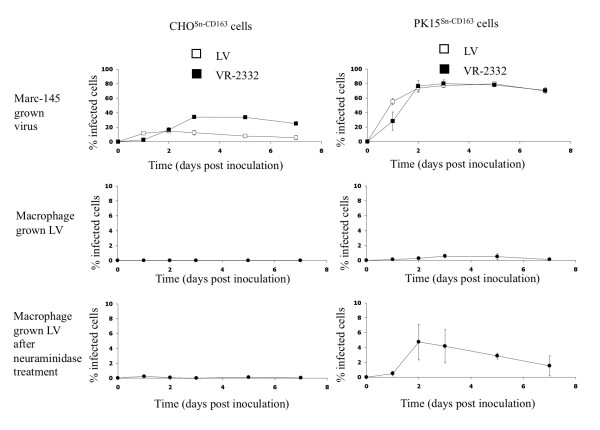
**PRRSV infection kinetics in CHO^Sn-CD163 ^and PK15^Sn-CD163 ^cells**. CHO^Sn-CD163 ^and PK15^Sn-CD163 ^cells were inoculated with Marc-145 grown LV (open square), Marc-145 grown VR-2332 (black square) or macrophage grown LV (black circle). After 1, 2, 3, 5 and 7 dpi the cells were fixed and an immunoperoxidase staining was performed. The amount of infected cells were counted and expressed in the graphs as the percentage of infected cells. Values represent mean ± SD of three experiments.

To investigate the susceptibility of CHO^Sn-CD163 ^and PK15^Sn-CD163 ^cells to PRRSV infection, cells were seeded at 200 000 cells/mL and infected with Marc-145 grown LV, Marc-145 grown VR-2332 or macrophage grown LV at a moi of 0.2 at 2 days post seeding. For macrophage grown virus infection, a comparison was made between infection of cells treated with neuraminidase and untreated cells. The cells were fixed 1, 2, 3, 5 and 7 dpi and stained by immunoperoxidase. Figure 4 shows that Marc-145 grown VR-2332 could infect more CHO^Sn-CD163 ^cells than Marc-145 grown LV, while both strains infect PK15^Sn-CD163 ^cells for approximately 80%. Macrophage grown virus did infect a low number of cells for the two cell lines (Figure 4). If the CHO^Sn-CD163 ^and PK15^Sn-CD163 ^cells were treated with neuraminidase, infection of the PK15^Sn-CD163 ^cells, but not the CHO^Sn-CD163 ^cells, with macrophage grown virus was increased (Figure 4). At 3 and 5 dpi, the highest amount of virus infection was achieved, with up to 20% for Marc-145 grown LV and up to 40% for Marc-145 grown VR-2332 on CHO^Sn-CD163 ^cells and up to 80% for both Marc-145 grown strains on PK15^Sn-CD163 ^cells. The infection rate of cells infected with macrophage grown virus did not reach 5% in both cell lines. After treatment of the cells with neuraminidase, infection with macrophage grown virus increased on PK15^Sn-CD163 ^cells. In summary, Marc-145 grown LV and Marc-145 grown VR-2332 could infect the cell lines most efficiently. Macrophage grown virus gave little infection, but if the cells were first treated with neuraminidase, infection was slightly better in the case of PK15^Sn-CD163 ^cells.

### Virus production in CHO^Sn-CD163 ^and PK15^Sn-CD163 ^cells

**Figure 5:**
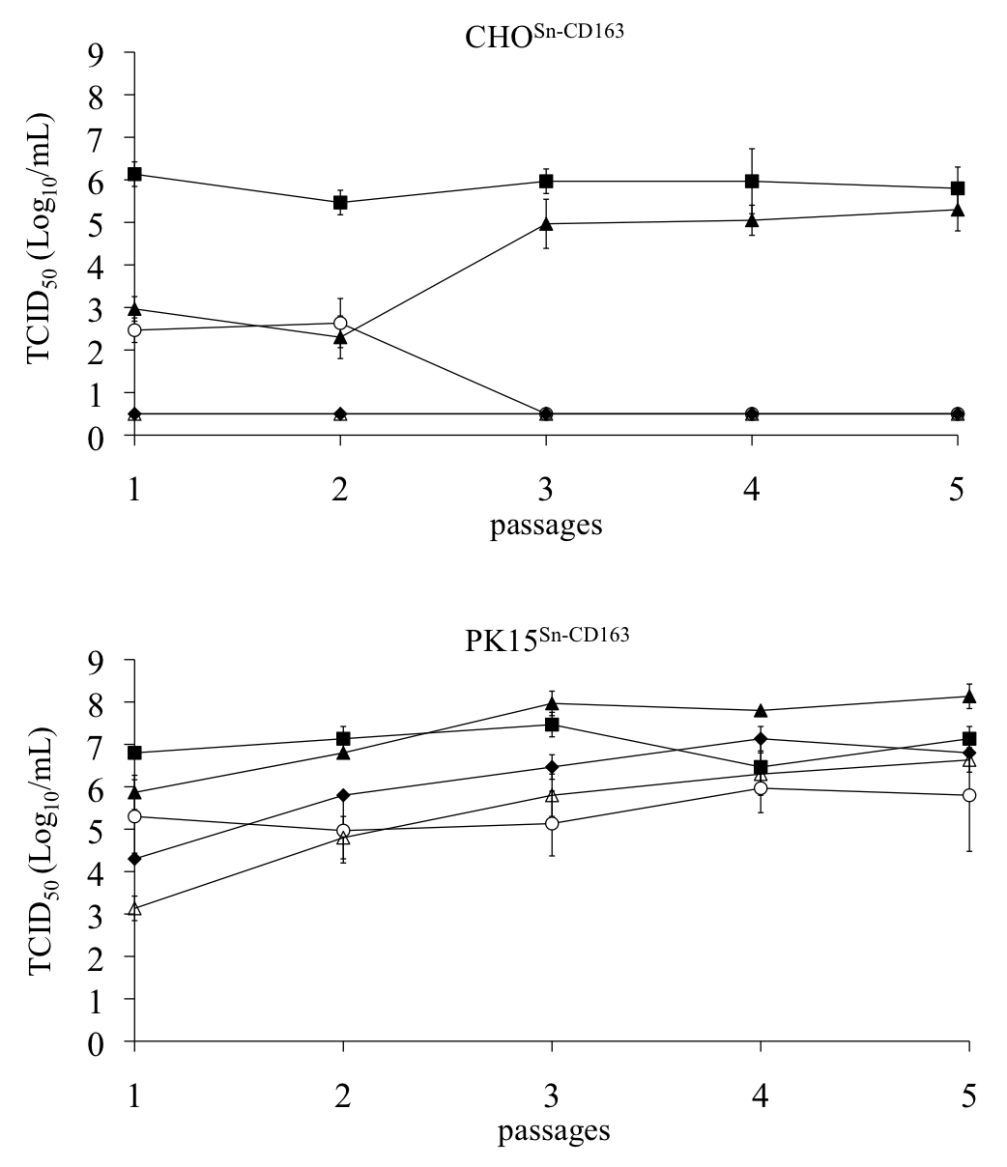
**Virus production on CHO^Sn-CD163 ^and PK15^Sn-CD163 ^cells**. CHO^Sn-CD163 ^clone IC5 and PK15^Sn-CD163 ^clone IXH7 were inoculated with Marc-145 grown VR-2332 (black square), macrophage grown LV (open circle), macrophage grown 07V063 (black triangle), macrophage grown 08V204 (open triangle) or macrophage grown 08V194 (black diamond). The virus was passages 5 times and the supernatant was titrated. Values represent mean ± SD of three titrations.

To determine virus production in CHO^Sn-CD163 ^and PK15^Sn-CD163 ^cells, the cells were inoculated with Marc-145 grown VR-2332, macrophage grown LV, macrophage grown 07V063, macrophage grown 08V204 and macrophage grown 08V194. When the virus was passaged for several times, the virus titer increased, especially in PK15^Sn-CD163 ^cells, to reach a stable level starting from passage 3. A titer of 10^6.0 ± 0.3 ^and 10^5.0 ± 0.6 ^TCID_50_/mL was obtained after 3 passages in CHO^Sn-CD163 ^cells for VR-2332 and 07V063 respectively. LV, 08V204 and 08V194 did not grow on CHO^Sn-CD163 ^cells. A titer of 10^5.1 ± 0.8^, 10^7.4 ± 0.3^, 10^8.0 ± 0.3^, 10^5.8 ± 0.5 ^and 10^6.5 ± 0.3 ^TCID_50_/mL was achieved after 3 passages in PK15^Sn-CD163 ^cells for LV, VR-2332, 07V063, 08V204 and 08V194 respectively. All virus titers of virus produced in both cell lines till passage 5 are represented in Figure 5. As a comparison, also virus yield obtained after 3 passages in Marc-145 cells was determined via titration on macrophages, revealing a titer of 10^6.6 ± 0.3^, 10^6.8 ± 0.6^, 10^7.1 ± 0.6^, 10^5.5 ± 0.6 ^and 10^7.1 ± 0.3 ^TCID_50_/mL for LV, VR-2332, 07V063, 08V204 and 08V194 respectively. Similar virus titers are thus produced in PK15^Sn-CD163 ^and Marc-145 cells.

### Virus sequencing after passaging virus in PK15^Sn-CD163 ^cells

As most virus strains grow better on PK15^Sn-CD163 ^cells, this cell line was more interesting to grow vaccine virus than CHO^Sn-CD163 ^cells. To produce vaccine virus for inactivated vaccines mutation of structural proteins should not occur. Therefore, PK15^Sn-CD163 ^grown virus was sequenced and compared to the sequence of the original virus. VR-2332 and 07V063 grown on PK15^Sn-CD163 ^cells showed no differences after 5 passages compared to the original virus strain. PK15^Sn-CD163 ^grown LV showed 1 amino acid (aa), 08V194 2 aa and 08V204 3 aa differences compared to macrophage grown virus. For LV the aa change was in ORF4 (N37D). The aa differences of 08V194 were located in ORF2 (E73D) and in ORF5 (N37S). For 08V204 the aa differences were situated in ORF2 (N37D), ORF4 (I121V) and ORF5 (N37D).

## Discussion

PRRSV replicates efficiently in ex vivo cultivated primary macrophages, which are the natural host cells. For vaccine virus production however, this cell type cannot be used, because of batch variation, risk of contamination with other pathogens and high production costs. PRRSV susceptible cell lines, such as the African green monkey derived cell lines, like Marc-145, have the potential to overcome problems associated with the use of primary macrophages, such as up-scaling and safety. However, PRRSV infects Marc-145 cells via a different entry pathway compared to macrophages, which results in adaptation of the virus for growth on Marc-145 cells [26].

Several non-permissive cells transfected with RNA of PRRSV could produce infectious virus. It is therefore suggested that the susceptibility of cells for PRRSV infection is determined by membrane-associated components [35]. In a previous study, it was shown that non-permissive cells transiently transfected with Sn only sustained internalization, but not infection [19]. Non-permissive cells transiently transfected with CD163 may allow a low level of infection depending on the cell type used [24]. Van Gorp et al. showed that co-expression of both Sn and scavenger receptor CD163 are needed for an efficient PRRSV infection [20]. A virus titer ranging between 10^2.4 ^and 10^5.5 ^TCID_50_/mL could be obtained upon PRRSV infection of cells transiently transfected with recombinant Sn and CD163 [20]. Since only a part of the cells were transfected upon transient transfection, it was expected that the virus titer would be higher if stably transfected cell lines could be used. In this study, cell lines that express both recombinant Sn and CD163 (CHO^Sn-CD163 ^and PK15^Sn-CD163^) were made, as both receptors are involved in infection of the natural host cell, the macrophage [20,27]. The constructed CHO^Sn-CD163 ^and PK15^Sn-CD163 ^cell lines were first analyzed for their PRRSV susceptibility. They were both susceptible for PRRSV, because LV and VR-2332 virus attachment, internalization, disassembly and infection occurred in both cell lines.

When analyzing the PRRSV susceptibility, it was shown that primary infection of the cell lines was more efficient with Marc-145 grown virus than macrophage grown virus. The infection rate of macrophage grown virus was very low, most likely because binding and internalization of macrophage grown virus particles into the cell lines was not efficient. The interaction of macrophage grown PRRSV with Sn, the receptor mediating binding to and internalization into the cells, is probably not efficient. This can be due to sialic acids present on the cells interfering with Sn, resulting in competition with sialic acid on PRRSV. This hypothesis is based on the observation that CD33, also a member of the sialoadhesin family, transfected in COS cells were not able to bind to red blood cells, containing sialic acid, unless the COS cells were first treated with sialidase to remove endogenous ligands [36,37]. Also CHO^Sn ^cells showed no binding of red blood cells unless they were treated with neuraminidase [32].

To investigate if neuraminidase could improve macrophage grown PRRSV infection, the CHO^Sn-CD163 ^and PK15^Sn-CD163 ^cells were first treated with *Vibrio cholerae *neuraminidase (Roche) to remove potential *cis*-acting sialic acids that could interfere with the sialic acid binding capacity of Sn [32]. This resulted in an increased amount of macrophage grown virus infected cells. These results suggest that the low virus titers are related with a low binding capacity of PRRSV to Sn, because of sialic acid present on the CHO^Sn-CD163 ^and PK15^Sn-CD163 ^cells. Marc-145 grown PRRSV will most likely also contain sialic acids that can interfere with Sn. Our hypothesis is that also the production of Marc-145 grown virus can be improved by neuraminidase treatment of the cells and needs to be further investigated.

However, using an expensive product like *Vibrio cholerae *neuraminidase is not ideal for vaccine production. To avoid this problem, virus is grown via several passages on the cell lines. Normally, titers of 10^5^-10^7 ^TCID_50_/mL can be obtained on Marc-145 cells after 5 to 7 passages [16,38]. The results show that the PK15^Sn-CD163 ^cells give similar results as a recent macrophage cell line transfected with CD163 [39]. In addition, the PK15^Sn-CD163 ^cells also express Sn, which is important to facilitate virus entry. The macrophage cell line transfected with CD163 also expresses Sn [39], which confirms that both receptors, Sn and CD163, are important for an efficient virus production. Virus growth on PK15^Sn-CD163 ^cells resulted in higher titers than growth on CHO^Sn-CD163 ^cells and the virus titer achieved on PK15^Sn-CD163 ^cells was equal to the titer on Marc-145 cells, which makes the PK15^Sn-CD163 ^cell line an interesting tool for virus production.

It is reported that due to adaptation of PRRSV to Marc-145 cells, mutations in non-structural, but also structural viral proteins may occur [40-42]. For the production of an inactivated virus vaccine, mutations in ORFs encoding viral structural proteins are not desired, since this can influence the induction of a virus neutralizing antibody response. The virus grown on PK15^Sn-CD163 ^cells is expected to show less mutations than after growth on Marc-145 cells, since the PK15^Sn-CD163 ^cells express Sn and CD163, two receptors important in the entry of the virus in macrophages and the entry-associated domains are probably involved in the induction of a neutralizing antibody response. To investigate if virus grown on PK15^Sn-CD163 ^cells showed mutation in ORFs encoding viral structural proteins, ORF2a, 3, 4, 5, 6 and 7 were sequenced. 07V063 and VR-2332 grown on PK15^Sn-CD163 ^cells showed a 100% aa identity with 07V063 grown on macrophages and VR-2332 grown on Marc-145 cells. LV grown on PK15^Sn-CD163 ^cells had 1 aa changed in ORF 4 compared to macrophage grown LV, which resulted in a loss of a putative glycosylation site. The mutation, however, was not situated in a known neutralizing epitope [43,44]. PK15^Sn-CD163 ^grown 08V194 had 2 aa difference compared to macrophage grown 08V194. There was 1 aa changed in ORF5. This resulted not in a loss of a glycosylation site, but the glycosylation site moved to another place. The mutation was not situated in a known neutralizing epitope [45]. The second aa change was located in ORF2a, however a change from E to D is supposed to have no effect on the protein structure since those aa are similar. For 08V204 grown on PK15^Sn-CD163 ^cells, there was 1 aa changed in ORF2a, 1 aa in ORF4 and 1 aa in ORF5. The mutation in ORF4, however was a change from I to V, which are comparable aa and will not have an influence on the protein structure. The mutation in ORF5 results in a loss of a putative glycosylation site, but is not located in a known neutralizing epitope [45]. The mutation in ORF5 of 08V194 and 08V204 are both on position 37. This position varies among different PRRSV strains and is a not well conserved glycosylation site [46]. These results indicate that the PK15^Sn-CD163 ^cells are useful for production of vaccine virus, but each strain should be investigated for aa changes in the structural proteins before use in inactivated vaccine production. Experiments are ongoing to test the immunogenicity of inactivated PRRSV grown on PK15^Sn-CD163 ^cells and the effect of the minor aa changes on the induction of a protective immunity towards challenge virus.

The observation that all tested strains grow well on the PK15^Sn-CD163 ^cells also suggests that these cells might be useful for virus isolation. Currently Marc-145 cells are used for diagnostics, but it has been shown that not all PRRSV strains can be detected on those cells [47]. Macrophages, the natural host cell of PRRSV, are more efficient for virus isolation [47]. The difference in isolation efficiency between Marc-145 cells and macrophages is suggesting that another receptor next to CD163 is involved in infection as Marc-145 cells and macrophages both express CD163 [20,24]. The PK15^Sn-CD163 ^cells can be useful for virus isolation, because of the expression of both CD163 and Sn, but needs to be further investigated.

## Conclusions

A PRRSV susceptible cell line expressing two major receptors for infection in macrophages, namely Sn and CD163, was constructed. It is shown that virus production from genotype 1 (LV, 3 Belgian strains) and genotype 2 (VR-2332) on the cell line is possible with virus titers being equal to the virus titers achieved on Marc-145 cells. No or only a few mutations occur in the ORFs encoding viral structural proteins and the mutations that occur for some viruses are not situated in known neutralizing epitopes. Therefore it is assumed that an inactivated PRRSV vaccine based on virus grown on the PK15^Sn-CD163 ^cells will be able to induce a proper immune response, but this is currently under investigation. In addition, the PK15^Sn-CD163 ^cells can probably also be used for virus isolation, because of the expression of both Sn and CD163, but this also needs to be confirmed.

## Authors' contributions

ID participated in the design of the experiments, performed the majority of the experiments, participated in the sequence alignments and wrote the manuscript. HVG carried out the cloning of CD163 into the pBUD plasmid. JVD carried out the sequencing of the virus strains and participated in the sequence alignments. PD and HN participated in the design of the experiments, supervised the study and critically revised the manuscript. All authors read and approved the final manuscript.
